# Pheochromocytoma With Brown Adipose Tissue Stimulation: A Case Report

**DOI:** 10.7759/cureus.54884

**Published:** 2024-02-25

**Authors:** Brayan Muñoz-Caicedo, Vanessa García-Gómez, Tatiana Arroyave-Peña, Alejandro Cardona-Palacio, Jack Muñoz-Caicedo

**Affiliations:** 1 Department of Radiology, Universidad de Antioquia, Medellín, COL; 2 Department of Radiology, Division of Body Imaging, Hospital Pablo Tobón Uribe, Medellín, COL; 3 Department of Radiology, Universidad Pontificia Bolivariana-CediMed, Medellín, COL; 4 Department of Pathology, Hospital Pablo Tobón Uribe, Medellín, COL

**Keywords:** pheochromocytomas, adrenal gland incidentaloma, brown adipose tissue, catecholamine cardiomyopathy, ct, mri, pet-ct

## Abstract

Brown adipose tissue represents about 1% of the adult body mass and decreases with age. Under variable circumstances, this amount changes, for example, with age or environmental conditions. Pathological states with hypersecretion of catecholamines can induce hypertrophy and hyperplasia in mature brown adipocytes. Consequently, this response can have imaging representation as pseudonodules, a pitfall in imaging interpretation, and may be confused with neoplastic involvement. A case of pheochromocytoma with brown fat stimulation and catecholamine cardiomyopathy is presented.

## Introduction

Adipocytes, in general, can be white or brown. They originate from different lineages with antagonistic functions: the whites for energy storage and the browns for heat production. Collectively, brown adipocytes make up the brown adipose tissue (BAT), which decreases with age, representing approximately 1% of the total body mass in adults. This tissue has typical or atypical locations. Despite being previously overlooked, recent research features the importance of BAT, shedding light on its regulatory roles in metabolism and thermodynamics. Other endocrine roles are out of the scope of this article [1-4]. BAT predominantly receives innervation from the noradrenergic sympathetic nervous system with a regulatory function stimulating the hypertrophy or hyperplasia of brown adipocytes or through the transdifferentiation of white to brown adipocytes. Consequently, BAT can have imaging representations as pseudotumors, and it is a cause of diagnostic error in imaging studies, for example, generating false-positive for malignancy in computed tomography (CT), magnetic resonance imaging (MRI), or fusion studies like positron emission tomography-CT (PET-CT) [5-8].

Its importance lies in recognizing that patients with increased production of catecholamine BAT may be more abundant with morphological changes in imaging. That is the case for patients with pheochromocytomas, neuroendocrine neoplasms of the adrenal gland medulla that, through catecholamines, trigger the response above, especially in locations close to the primary tumor [5,6].

The case of a patient with a final diagnosis of pheochromocytoma with stimulation of perinephric brown fat and catecholamine cardiomyopathy is presented below.

## Case presentation

**Figure 1:**
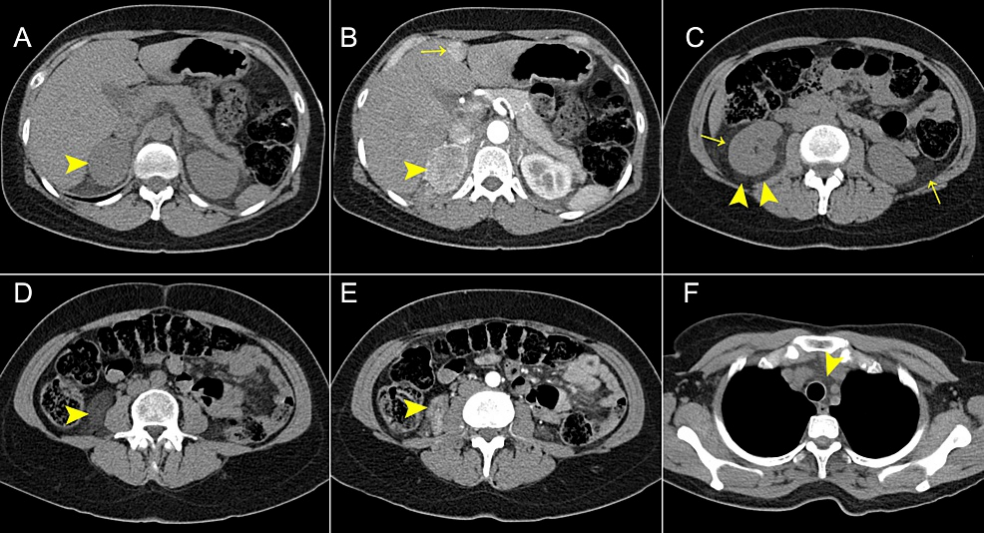
CTA of the thoracoabdominal aorta. (A) The NECT axial plane shows a right-sided, round, and well-defined adrenal mass (➤) without calcifications or fat areas. The mass measured 41 x 46 x 34 mm. (B) In the CECT arterial phase, the adrenal mass showed avid and heterogeneous enhancement up to 145 HU (➤). Incidental hypervascular liver nodules were seen (→). (C) NECT of the perinephric bilateral spaces with striated fat (→) and pseudonodular lesions (➤), mainly to the right side. (D) Below the right renal pole was the bigger pseudolesion that measured 21 x 44 x 32 mm (➤) and had attenuation rounding of -51 HU in the NECT. (E) This nodule had enhancement with the contrast agent (➤). (F) NECT with striated fat in the mediastinum (➤). The measurement units are in millimeters (mm) and are in order length x anteroposterior x transverse. CTA: computed tomography angiography; NECT: non-enhanced computed tomography; CECT: contrast-enhanced computed tomography; HU: Hounsfield unit.

A 47-year-old woman with a history of high blood pressure consulted the emergency department for one hour of chest pain and nausea. Acute aortic syndrome was suspected, and an emerging CT angiography (CTA) of the thoracoabdominal aorta was initially performed. The CT ruled out this diagnosis. However, a right adrenal mass with adjacent pseudo-nodular and fat-stranding changes and hypervascular liver lesions were characterized (Figure 1). The pheochromocytoma, paragangliomas, and Castleman disease were in the imaging differential diagnoses.

Cardiovascular causes were also initially considered. The electrocardiogram was normal; nonetheless, troponins were positive for myocardial injury, and an acute myocardial infarction without ST-segment elevation was diagnosed. Coronary angiography revealed no significant lesions. In the echocardiogram, akinesia and bulging of all apical walls and hypokinesia of the mid-segments were seen. The findings indicated stress cardiomyopathy.

**Figure 2:**
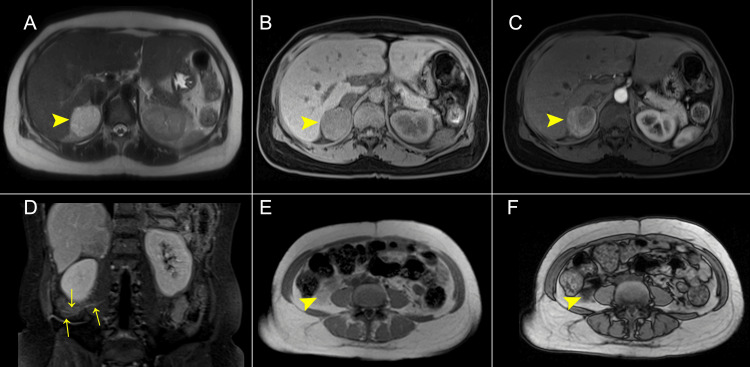
Gadolinium-enhanced abdominal MRI with non-hepatic-specific contrast. (A) T2WI sequence axial plane with markedly hyperintense signal of the right adrenal mass (light bulb sign) and (B) T1WI hypointensity signal. (C) T1FS post-contrast arterial phase axial imaging with hypervascular behavior. (D) Prominent vascular structures were present. (E) Dual-echo in-phase and (F) opposed-phase T1-weighted imaging showing loss of signal of the lesion inferior to the right renal pole. MRI: magnetic resonance imaging; T1WI: T1-weighted imaging; T1FS: T1 fat suppression; T2WI: T2-weighted imaging.

Upon further questioning, the patient reported several weeks of headaches, paresthesia in the extremities associated with episodes of palpitations, and facial redness. Malignant pheochromocytoma or paragangliomas were in the differential diagnoses, so the findings were further characterized by contrast-enhanced MRI of the abdomen (Figure 2).

The abdominal MRI reported findings consistent with right adrenal pheochromocytoma with stimulation of the perinephric brown fat and focal nodular hyperplasia in the liver without findings suspicious of metastasis.

**Figure 3:**
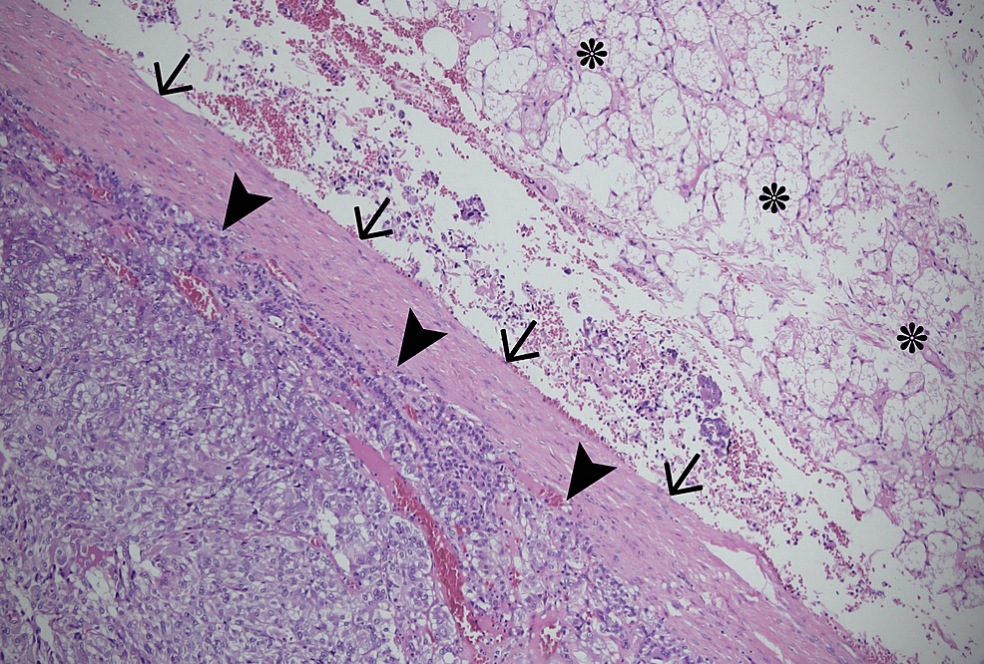
Histopathological finding pheochromocytoma. Hematoxylin-eosin stain, original magnification 40x. In the right adrenal gland, a well-circumscribed lesion (➤) delimited by a fibrous capsule (→) and without extra-adrenal invasion (negative surgical margins). Brown fat (*) is visible surrounding the adrenal gland. The Pheochromocytoma of the Adrenal Gland Scaled Score (PASS) was 1 point.

The laboratories reported normal fractionated metanephrine levels in the 24-hour urine sample, with plasmatic free metanephrines at 169 pg/mL (reference: 0-90), free normetanephrine at 2836 pg/mL (reference: 0-180), and total metanephrines at 3005 (reference: 0-270). The aldosterone lab value was normal. Plasma renin activity was 12.17 ng/ml/h (reference: 0.04-4.95). Subsequently, the patient received pre-surgical α and β blockade titrated according to the clinical response and later laparoscopic right adrenalectomy with a final pathological diagnosis of pheochromocytoma surrounded by brown fat with inflammatory changes (Figure 3).

**Figure 4:**
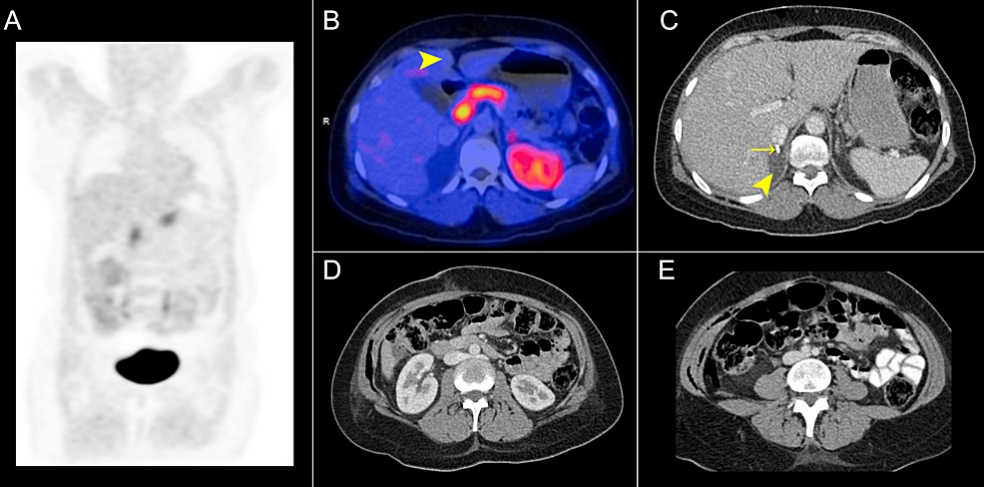
18F-DOPA PET-CT in the early post-adrenalectomy. (A) Coronal projection at the maximum intensity of the ^18^F-DOPA PET-CT without suspicious metastasis or tumor viability uptake areas. (B) The liver lesions did not uptake the molecule. (C) Axial CECT image with a hypodense seroma in the surgical bed of the right adrenalectomy (➤), surgical clips (→), and postoperative emphysema, normal findings for the time of evolution. (D) The perinephric and (E) right inferior renal pole pseudonodular lesions visualized on the diagnostic CT disappeared. ^18^F-DOPA PET-CT: ^18^F-dihydroxyphenylalanine PET-CT; CECT: contrast-enhanced computed tomography.

The patient had a postoperative course with a seroma in the surgical bed and had no other complications. In the 18F-dihydroxyphenylalanine (18F-DOPA) PET-CT (Figure 4), no lesions showing tumor viability or metastasis were identified with a complete response to surgical treatment. The morphological pseudotumoral appearance in the perinephric spaces also disappeared. The genetic analyses of the patient, such as GDNF, KIF1B, FH, MAX, MEN1, NF1, RET, SDHA, SDHAF2, SDHB, SDHC, SDHD, TMEM127, and VHL, were negative.

## Discussion

Myf5+ progenitor cells differentiate to give rise to brown adipocytes, the primary function of which is heat production. The cytoplasm of brown adipocytes holds lipid droplets of smaller size but greater number, making them more hydrated than white adipocytes. Another particularity of the former is the increased mitochondria, organelles rich in iron that confer a brown color to the tissue under natural light [8]. BAT is a normal tissue that gradually decreases over time, reaching approximately 1% of the total body mass in adults. It has a regulatory function in metabolism and thermodynamics. In adults, the location tends to be cervical, supraclavicular, axillary, paravertebral, intercostal, mediastinal, retroperitoneal, and abdominal wall. Atypical locations are in the posterior aspect of the neck, left paratracheal area, axillary, perinephric, retrocrural, and mesentery [2,9].

Sympathetic nerve endings release norepinephrine, stimulating the β-3 adrenoceptors of mature brown adipocytes. This has a regulatory function by increasing the number of brown fat cells, lipolysis, glucose transport, and uncoupling protein-1 (UCP-1) expression, resulting in heat production [1,10]. Persistently elevated catecholamines lead to upregulation of UCP-1, triggering BAT hyperplasia and hypertrophy and inducing transdifferentiation of white adipocytes to brown adipocyte-like cells, generating a pseudotumoral appearance, especially in locations close to the pheochromocytoma [11]. Here lies the importance of knowing this potential pitfall interpretation as metastasis or hematological malignancies in morphological and metabolic studies like CT, MRI, or PET-CT [1,5,6,11].

Identification of BAT in imaging requires suspicious areas related to low attenuation on the CT scans or the coregistered CT in PET with Hounsfield unit (HU) of fat density (range: -250 to -50 HU), size greater than 4 mm, and a maximum standardized uptake value of 18F-fluorodeoxyglucose (18F-FDG) of at least 2.0 g/mL. Typical locations and bilateral findings support the diagnosis [7,12,13]. The MRI technique takes advantage of the fact that BAT has a higher water content. In that environment, the protons' precession differs from the surrounding white adipose tissue (WAT), with higher magnetic susceptibility. In other words, the signal intensity varies as a function of water content, with approaches using chemical shift imaging, spectroscopy, and multi-echo Dixon-based pulse sequences. The mitochondrial iron, in the susceptibility gradients, generates an effect visible in T2* sequences and T2-weighted images where BAT is hypointense compared with WAT. Other advanced MRI sequences, for example spectroscopy, have been described [8,12,14,15,16].

Differential diagnoses of the stimulated and hypertrophied BAT include malignant pheochromocytoma with retroperitoneal spread, renal carcinoma with adjacent extension, perirenal metastases (melanoma, lung, or breast cancer), extramedullary hematopoiesis, Erdheim-Chester, retroperitoneal fibrosis, lymphoma, and sarcoma, among other possibilities.

On the other hand, pheochromocytomas are rare neuroendocrine neoplasms whose clinical presentation has been changing with imaging access. When patients diagnosed in screening due to genetic predisposition are excluded, those diagnosed with pheochromocytomas from the workup of adrenal incidentalomas (AI) represent 69%, followed by presentation of adrenergic symptoms in 28% and acute cardiomyopathy in 3%. Conversely, of all AI, 7% are pheochromocytomas. For this reason, patients with AI are recommended to be evaluated with metanephrine measurements in plasma and urine [5,17].

Pheochromocytoma is suspected in patients with symptoms and signs of catecholamine excess, poorly controlled or paroxysmal hypertension, adrenergic symptoms, or the classic triad of headache, sweating, and palpitations. This triad is highly specific, around 90%, but present only in 10-36% of the cases. Of the patients with AI with a final diagnosis of pheochromocytoma, up to 41% retrospectively reported typical symptoms. Undiagnosed cases relate to mortality likely due to cardiovascular causes. Malignant pheochromocytomas defined for metastases are rare, present with adrenergic symptoms, and have clear dissemination in imaging studies [5,6,11,17].

In the study by Aggarwal et al. [5] with 167 patients with pheochromocytomas, 48% of the cases were right-sided, 45% left-sided, and 7% bilateral, and metastases were found in 2.4%. The average size was 45 mm (range: 10-215 mm). However, subgroups diagnosed in the screening for susceptibility vs. incidental vs. symptomatic had sizes of 30 mm vs. 42 mm vs. 60 mm, respectively. In non-enhanced computed tomography (NECT), pheochromocytomas have indeterminate characteristics, a heterogeneous appearance, or attenuation of more than 10 HU. No case of pheochromocytoma had a homogeneous density of less than or equal to 10 HU. However, rare pheochromocytomas with fat can have less than 10 UH and emulate adenomas [17]. With contrast administration, there is brisk enhancement of the solid components and washout that may be similar to adenomas or malignant metastatic adrenal lesions [17,18].

MRI characteristics are variable and depend on the hemorrhagic, cystic, or necrotic changes. The classic appearance is the hyperintensity on the T2WI sequence described as a “light bulb,” and the solid part tends to be hypervascular in the dynamic sequences. The appearance of MRI sequences T1 and T1 in-phase and opposed-phase varies with the changes in the mass [17,18]. PET-CT studies with F-DOPA, 18F-deoxyglucose, or 18F-fluorodopamine have better sensitivity than metaiodobenzylguanidine scans, especially for the evaluation of metastatic pheochromocytoma [19]. Nuclear medicine studies are particularly important in studying extra-adrenal paragangliomas, metastatic, or recurrent disease [20].

The laboratory studies can include measurement of fractionated metanephrines in plasma or 24-hour urine, with the former being the most sensitive [17]. Mean metanephrine levels tend to be much higher in patients with adrenergic symptoms or uncontrolled hypertension than in those with AI (15-fold versus five-fold elevation above the normal upper limit) and correlate with the tumor size [5].

Management includes symptomatic pharmacologic control and laparoscopic adrenalectomy for most pheochromocytomas. Open surgery in cases with large masses (>6 cm) or invasive features is indicated [20]. Pathologic macroscopic features are well-circumscribed masses with variable color, mainly yellow, but hemorrhagic, necrotic, or cystic changes may be visible. The weight documented was up to 4 kg. The microscopic analysis reveals especially chromaffin and sporadically spindle cells. The Pheochromocytoma of the Adrenal Gland Scaled Score (PASS) is a score composed of 12 histologic characteristics that result in a score of 4 or more, denoting an aggressive clinical course, while those with PASS ≤ 3 have reported evidence of no metastasis until 14 years of follow-up [17].

Genetic studies after diagnosing pheochromocytoma are a shared decision-making process with patients [20]. They have shown mutations in NF1 (28%), RET (19%), SDBH (19%), and VHL (14%), among others. Incidental pheochromocytoma tends to be less likely to be hereditary [5]. Risk factors for hereditary cases are age less than 25 years and bilateral tumors. The prognosis is good for patients with surgical excision of nonmetastatic neoplasms, achieving a cure. The main risks are incomplete tumor resection or spillage during surgery. Tumoral recurrences occur in the surgical bed or as metastases [17,20].

## Conclusions

This case presents a patient with a catecholamine-producing pheochromocytoma with adjacent pseudolesions of infiltrative appearance explained by brown fat hypertrophy as the most probable diagnosis prospectively. The free margins of adrenalectomy, post-surgical resolution of the finding, and the absence of uptake in the F-DOPA PET-CT corroborate the diagnosis. Additionally, it is noteworthy that pheochromocytoma is increasingly diagnosed in the workup of adrenal incidentalomas, likely related to the accessibility of imaging studies. This favors the recommendation that adrenal incidentalomas be evaluated for pheochromocytoma, except in asymptomatic patients and tomographic characteristics of benign adenomas.
